# Pre-Scheduled and Self Organized Sleep-Scheduling Algorithms for Efficient *K*-Coverage in Wireless Sensor Networks

**DOI:** 10.3390/s17122945

**Published:** 2017-12-19

**Authors:** Prasan Kumar Sahoo, Hiren Kumar Thakkar, I-Shyan Hwang

**Affiliations:** 1 Department of Computer Science and Information Engineering, Chang Gung University, Guishan, Taoyuan 33302, Taiwan; pksahoo@mail.cgu.edu.tw (P.K.S.); D0221015@stmail.cgu.edu.tw (H.K.T.); 2 Division of Cardiology, Department of Internal Medicine, Chang Gung Memorial Hospital, Linkou, Taoyuan 33305, Taiwan; 3 Department of Computer Science and Engineering, Yuan Ze University, Chung-Li District, Taoyuan 32003, Taiwan

**Keywords:** wireless sensor networks, scheduling, K-coverage, energy efficient

## Abstract

The *K*-coverage configuration that guarantees coverage of each location by at least *K* sensors is highly popular and is extensively used to monitor diversified applications in wireless sensor networks. Long network lifetime and high detection quality are the essentials of such *K*-covered sleep-scheduling algorithms. However, the existing sleep-scheduling algorithms either cause high cost or cannot preserve the detection quality effectively. In this paper, the Pre-Scheduling-based *K*-coverage Group Scheduling (PSKGS) and Self-Organized *K*-coverage Scheduling (SKS) algorithms are proposed to settle the problems in the existing sleep-scheduling algorithms. Simulation results show that our pre-scheduled-based KGS approach enhances the detection quality and network lifetime, whereas the self-organized-based SKS algorithm minimizes the computation and communication cost of the nodes and thereby is energy efficient. Besides, SKS outperforms PSKGS in terms of network lifetime and detection quality as it is self-organized.

## 1. Introduction

In the recent past, numerous diversified applications of Wireless Sensor Networks (WSNs) had been explored to carry out coordinated tasks for demanding applications such as intruder detection [1], fire detection, health monitoring [2], etc. In WSN, normally each sensor first carries out the local monitoring and transmits the results to a designated base station, which combines the set of local monitoring and generates a global status of the area of interest. It is observed that sensor nodes are unreliable in their individual capacity to cover the monitoring region, and therefore, it is highly desirable to maintain a high degree of coverage to minimize the effect of malfunctioning sensors and to enhance the detection quality [3].

There are two broader categories of approaches such as pre-scheduled and self-organized, based on which the existing sleep scheduling algorithms can be classified. In the pre-scheduled approach [4,5], the entire monitoring duration consists of the Initialization Phase (IP) followed by a long Sensing Phase (SP), where SP is again composed of several rounds of equal time duration. In the IP, each sensor first establishes a working schedule and then performs the duty cycle continuously. Since, the working schedule of each sensor is maintained by the sink, the *K*-coverage configuration maintenance cost can be reduced significantly. However, the reduction in maintenance of cost comes with an extra burden of precise time synchronization among the neighbors in the long SP. Moreover, when some sensor runs out of battery or fails accidentally, the pre-defined scheduling on each sensor may need to be updated. Hence, the overall cost of performing the pre-scheduled approach could be still high.

To address the problems existing in the pre-scheduled approach, numerous self-organized *K*-coverage eligibility algorithms [6,7] are designed to be executed in a periodic manner. The monitoring operation of this approach is composed of several rounds, with each round consisting of IP and SP. Each round starts with an IP, followed by a SP. In the IP, an eligibility of each sensor is verified to determine whether sensor will stay active in the SP or not. In comparison with the pre-scheduled approach, the self-organized approach is superior in terms of minimizing the coverage redundancy of the *K*-coverage configuration. Besides, the self-organized approach dynamically chooses the sensors with better conditions (e.g., sufficient battery or location) to preserve the detection quality. Several studies [8] indicate that system false positive (false alarm) and system false negative (detection miss) are two major performance metrics in WSNs For the detection quality. However, the computational cost of the existing well-known algorithms [6,7] is too high to perform long-term monitoring.

It is observed that both pre-scheduled and self-organized approaches are widely used to perform the *K*-coverage configuration. From the study of numerous literature works, it is found that a very limited number of works analyze the overall performance of these two approaches. For monitoring critical applications in WSNs, the detection quality and the network system lifetime are the two most important issues, especially under the *K*-coverage configuration deployment. We argue that the detection performance of the *K*-coverage configuration may not be guaranteed by just increasing the value of *K* simply. Hence, in this paper, the Pre-Scheduling-based *K*-coverage Group Scheduling (PSKGS) and Self-Organized *K*-coverage Scheduling (SKS) algorithms are proposed to settle the problems existing in the pre-scheduled and self-organized approaches, respectively. The main contributions of our work can be summarized as follows.

- Novel PSKGS and SKS algorithms are designed to improve the performance of the pre-scheduled and self-organized approaches of WSNs, respectively.
- Detection performance of the *K*-coverage sleep scheduling is measured in terms of explicit metrics introduced in [8], i.e., by using the probability of system false positives and system false negatives.
- The network lifetime of PSKGS and SKS is evaluated, and the impact of the sleep-scheduling algorithms on the overall detection quality is analyzed.
- Though both pre-scheduled and self-organized approaches are widely used, the proposed work analyzes the suitability of self-organized and pre-scheduling approaches to guarantee the *K*-coverage configuration while maximizing the network lifetime.

The rest of the paper is organized as follows. Related works of pre-scheduling and self-organized approaches are discussed in Section 2. The background and problem analysis is introduced in Section 2.1. The PSKGS algorithm with enhancement of the pre-scheduled approach is described in Section 3. The SKS with enhancement of the self-organized approach is illustrated in Section 4. Performance evaluation of both approaches is given in Section 5, and concluding remarks are made in Section 6.

## 2. Related Works

As we know, scheduling of nodes plays a vital role in improving the network system lifetime. Besides, the network lifetime of the nodes can be increased by scheduling the nodes based on their degree of coverage. Designing an efficient *K*-coverage configuration covering each area of the monitoring region using *K* number of Active Sensors (ASs) has attracted much attention in the recent past. The authors in [9] have proposed a mechanism to monitor the event coverage in WSNs using dynamic adjustment of sensing range. In [10], a *K*-coverage model based on the genetic algorithm is designed to extend the lifetime of WSN. The authors in [11] employ the probabilistic sensing model and calculate the least number of sensor nodes that is required in a randomly-deployed WSN for target coverage. The problem of determining the probability of a sensor being redundant for the *K*-coverage of the field of interest is discussed in [12], and the coverage and connectivity issues of WSNs are discussed in [13,14]. The authors in [15,16] measure the fault tolerance of the *K*-coverage configuration. Several algorithms for the sleep scheduling mechanism have been designed to preserve the coverage degree while maximizing the system lifetime [17,18,19,20]. Identifying the optimal deployment locations of the sensors and scheduling based on such knowledge with a pre-defined sensing range is discussed in [21] for the network lifetime maximization with the required coverage level.

A distributed localization protocol for WSN is proposed in [22], where sensors can cooperate with each other to find the relative location of the nodes to estimate the coverage of the nodes. However, the authors do not describe how nodes should collaborate to maintain the coverage. Though centralized and distributed connected target *K*-coverage algorithms for the heterogeneous wireless sensor networks are proposed in [23], there is no scheduling mechanism to improve the network lifetime and to maintain the target coverage. A genetic algorithm-based sensor deployment algorithm is designed in [24] to increase the coverage of the homogeneous WSN. Considering a heterogeneous WSN with variable sensing and communication radii of the sensors, a scheduling mechanism is designed in [25] to identify the redundant sensors. Recently, people have tried to address the design issues of homogeneous WSNs for the heterogeneous network environment to provide advanced services including topology maintenance [26], deployment and placement, energy-efficient clustering [27], time synchronization and sleep scheduling [25]. In [28], the limited mobility coverage and connectivity maintenance protocols are discussed for wireless sensor networks.

The authors in [29] have designed protocols to deploy mobile sensors to provide the target coverage and network connectivity with the requirements of moving sensors. In order to estimate the probabilistic area coverage in WSN, the authors in [30] find the relationship between the coverage of two adjacent points and transform the full area coverage problem into a point coverage problem for the *K*-coverage configuration. In [31], the *K*-coverage problem of WSN is handled by considering the Coverage Contribution Area (CCA) as a parameter to prolong the network lifetime. In [32], the network connectivity of the *K*-coverage sensors is addressed, and the *K*-coverage scheduling protocol is designed to ensure that at least *K* number of active sensors stays connected in each round. In [33], a distributed coverage hole repair algorithm for wireless sensor networks is proposed considering the density of the nodes in the post-deployment scenario.

In order to realize full coverage and *K*-connectivity in WSN, the authors in [34] have developed a deployment strategy using a genetic algorithm to monitor crops. However, the work does not discuss the node scheduling for the power constraint wireless sensors. A coverage-preserving node-scheduling scheme for WSNs is proposed in [35] to schedule the sensors in sleep and active mode. However, there is no self-organized approach to maintain target *K*-coverage in this work. In this section, we briefly introduce the existing well-known pre-scheduled and self-organized approaches, which help us to understand the enhancement of the proposed PSKGS and SKS algorithms given in the next subsequent sections.

### 2.1. Background and Problem Analysis

In this sub-section, a brief background of pre-schedule and self-scheduled approaches is provided to introduce their fundamental procedures. Besides, this sub-section also discusses the shortcomings of the existing state-of-the-art pre-scheduled and self-scheduled algorithms and the scope for further improvement.

#### 2.1.1. Pre-Scheduled Approach

**Figure 1:**
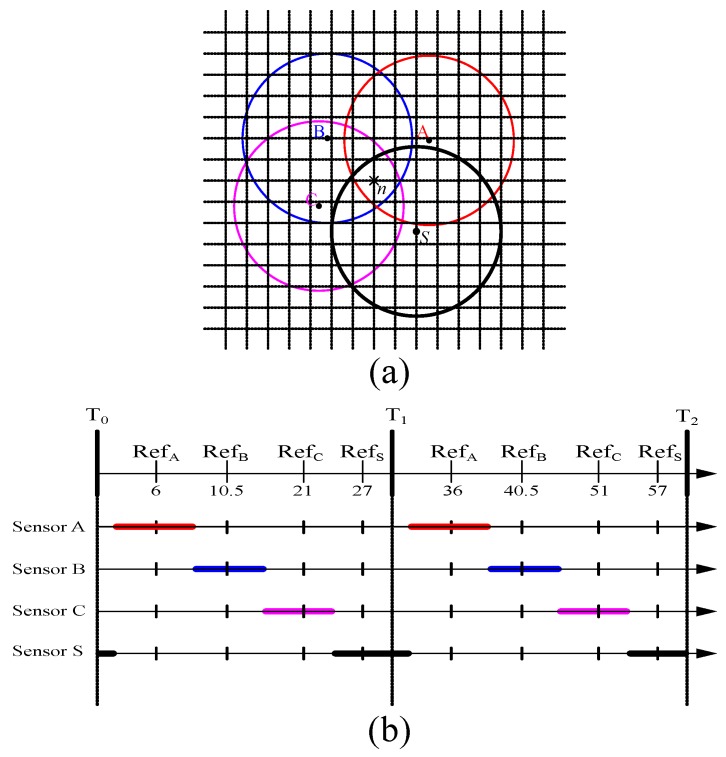
An illustration of the pre-scheduled grid-based algorithm. (**a**) The division of sensing range of sensor *s* into multiple Grid Points (GPs), in which grid point *n* is also covered by sensors *A*, *B* and *C*. (**b**) Sensors *A*, *B*, *C* and *S* establish the duty cycle for grid point *n*.

The pre-scheduled approach minimizes the K-coverage configuration maintenance cost. Normally, in the pre-scheduled-based algorithms, the monitoring period consists of two phases called the Initialization Phase (IP) and the Sensing Phase (SP). The task of establishing duty cycles usually takes place in the IP. In order to schedule all sensors, the sensing range of each sensor is divided into several virtual square grids as discussed in [5]. To conserve the energy expenditure, sensors are scheduled in such a way that whenever any grid point is found covered by numerous sensors, all of the participating sensors wake up one after another to monitor the point. Taking Figure 1a as an example, grid point *n* is located inside the sensing range of sensor *s*, and at the same time, it is also covered by sensors *A*, *B* and *C*. To balance the power consumption of these sensors, sensors *A*, *B*, *C* and *S* should wake up to monitor the grid point *n* one after another without overlapping their active periods. As shown in Figure 1b, let us consider that each round $T_{r}\in \left\{\left\{T_{1}-T_{0}\right\},\left\{T_{2}-T_{1}\right\}\right\}$ of a sensing phase consists of uniform time duration of $T_{r}=30$ s. Since, four sensors *A*, *B*, *C* and *S* participate to cover the grid point *n*, each sensor randomly chooses a reference time instance $ref_{A}$, $ref_{B}$, $ref_{C}$ and $ref_{S}$ in each round of $T_{r}$. As shown in Figure 1b, for round $T_{r}=\left\{T_{1}-T_{0}\right\}$ and $T_{r}=\left\{T_{2}-T_{1}\right\}$, sensors *A*, *B*, *C* and *S* are randomly chosen for the reference time instance $Ref_{A}=06$, $Ref_{B}=10.5$, $Ref_{C}=21$ and $Ref_{S}=27$, and $Ref_{A}=36$, $Ref_{B}=40.5$, $Ref_{C}=51$ and $Ref_{S}=57$, respectively. 

**Figure 2:**
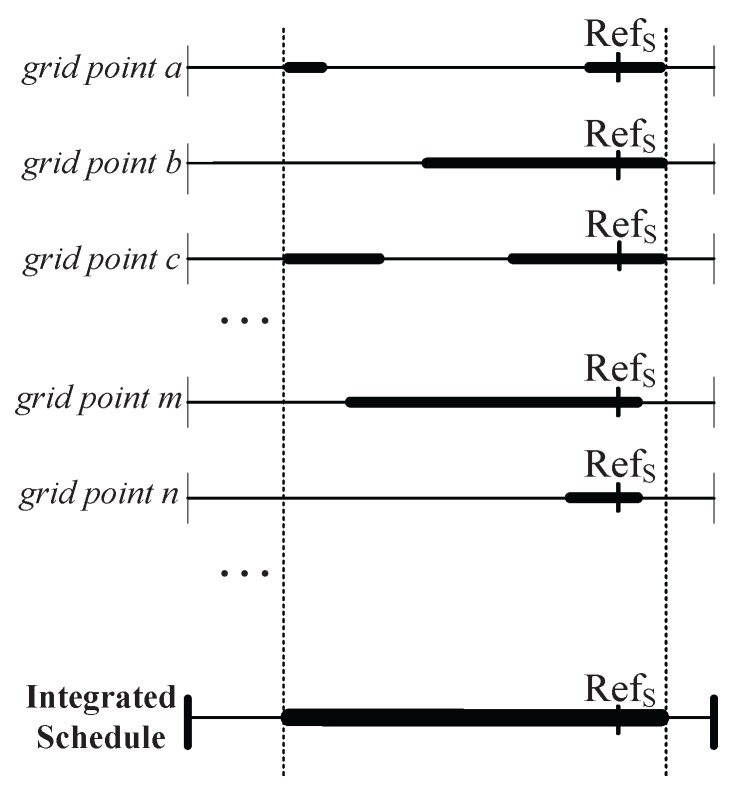
The duty cycle of sensor *S* is integrated from the scheduling of all GPs located within the sensing range of sensor *S*.

Normally, in the sensing range of an individual sensor, numerous GPs may exist, which are covered by various neighbors. For that reason, the sensor needs to be active for a longer duration to satisfy all working schedules of these GPs as shown in Figure 2. Consequently, the overall coverage degree is highly increased with many redundant Active Sensors (ASs) due to inappropriate scheduling granularity. On the other hand, if the size of the virtual grids is large, the generated sleep scheduling for all sensors may not guarantee the *K*-coverage configuration. As described in [5], how to define the size of the virtual grids is a significant issue as the algorithm itself has a tradeoff between the detection quality and system lifetime.

In [4], the authors establish the working schedule of each sensor based on the Intersection Points (InPts) rather than the Grid Points (GPs) within the sensing range of a sensor. It is proven that a sensor is *K*-covered [6], if all InPts generated by the sensing ranges of the neighbors of a sensor are located within the sensing range of this sensor. Compared to GPs, the number of InPts is minuscule in a sparse wireless sensor network. Hence, the intersection-based algorithm [4] effectively reduces the overall coverage degree. However, the intersection-based algorithm suffers from cubic time complexity represented as $O(n^{3})$, where *n* represents the number of neighbors of a sensor. It is observed that within the sensing range of a sensor, the number of InPts increases and decreases with the increase and decrease of node density, respectively.

To satisfy all schedules of the InPts within the sensing range, a greater number of redundant active sensors needs to stay active, which increases the probability of system false alarms due to severe signal interference among those sensors. For the pre-scheduled approach, the scheduling granularity adopted in both grid-based [5] and intersection-based algorithm [4] should be improved. In the proposed PSKGS, a sensor establishes the duty cycle based on its own neighbors, which are divided into several one-covered groups with minimum overlapping. Hence, the overall coverage degree is effectively reduced while guaranteeing the coverage.

#### 2.1.2. Self-Organized Approach

As discussed above, it is to be noted that the pre-scheduled approach has a higher coverage degree as all sensors establish their own sleep schedule only once during the whole monitoring period. However, in the pre-scheduled approach, the sleep schedule of many sensors cannot be optimized. In contrast, the monitoring period in the self-organized approach is divided into rounds with equal duration. Each round has an IP and an SP. In each round, sensors with a better condition such as energy level or detection quality can be chosen to monitor the area. Hence, the detection quality of the self-organized approach may be better than that of the pre-scheduled approach.

In [6], it is established that a region under monitoring is called *K*-covered, when all of the InPts are *K*-covered. Hence, in [6], a Coverage Configuration Protocol (CCP) is proposed to cover *K* nodes. In CCP [6], it is decided either to keep the sensor active or send it to sleep mode by tracing all of the InPts for each sensor. A sensor is qualified to go to the sleep state if the InPts within its sensing range have a coverage degree higher than *K*. However, CCP [6] also suffers from cubic time complexity $O(n^{3})$, where *n* represents the number of neighbors of a sensor. In a dense network, the cubic time computational complexity of CCP [6] may result in excessive power consumption, which subsequently makes it very difficult to carry out long-term monitoring of the low powered wireless sensors. Hence, the *K*-Perimeter-Covered (KPC) algorithm [7] is proposed, where the eligibility of each sensor is decided by tracing only the perimeter coverage. A sensor becomes a candidate for going to sleep if its perimeter is *K*-covered by its neighbors. The complexity of this procedure in KPC [7] is effectively reduced to O(nlogn).

It is to be noted that KPC [7] cannot calculate the coverage degree of a sensor correctly based only on its own perimeter coverage. However, a sensor must get some information from its neighbors to calculate its coverage degree. Hence, the complexity of KPC [7] on each sensor may be higher than that of CCP [6] instead, due to extra communication cost. In [36], though the authors propose distributed energy-efficient *K*-coverage eligibility and *K*-group scheduling algorithms, there is no analysis of network system lifetime and the impact of the sleep scheduling algorithms on the overall detection quality. Besides, the scope of performance evaluation of the proposed protocols is very limited. Quality of surveillance on the *K*-coverage configuration is analyzed in [37]. However, no scheduling mechanism is proposed to improve the network lifetime and to maintain the required *K*-coverage simultaneously. Hence, we develop here energy-efficient scheduling mechanisms with extensive simulation results to study *K*-coverage configurations in WSNs. Unlike CCP [6] and KPC [7], our proposed SKS focuses on discovering the candidate regions with lower coverage degree within the sensing range of each sensor. Instead of performing redundant tracing of all InPts that lie within the sensing range of a sensor, SKS only traces those InPts encircling the candidate regions. In turn, SKS greatly reduces the complexity and at the same time assures the *K*-coverage configuration.

## 3. Pre-Scheduling Based *K*-Coverage Group Scheduling

**Figure 3:**
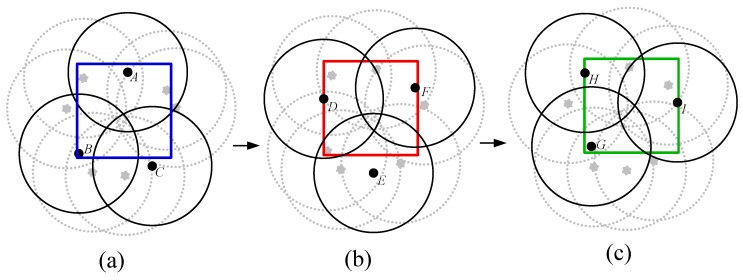
An illustration of PSKGS sleep scheduling for *K* = 1 by group of sensors. (**a**) *A*, *B*, and *C*; (**b**) *D*, *E*, and *F*; (**c**) *H*, *I*, and *G*.

In order to address the problems of the current pre-scheduling approaches [4,5], our proposed *K*-coverage Group Scheduling (PSKGS) algorithm schedules sensors in a group, where each sensor in a group works for an equal duration by turns leading to balancing of the overall energy expenditure of the sensors. Taking Figure 3 as an example, sensors *A*, *B* and *C* are grouped to monitor the square area for *K* = 1 (Figure 3a); sensors *D*, *E* and *F* (Figure 3b) and sensors *H*, *G* and *I* (Figure 3c) form different groups to monitor the area by turns. PSKGS has two unique qualities. (1) The deployed sensors are clustered into groups in such a way as to ensure members of any group are one-covered and, at the same time, so as to ensure the least overlapping among group members. (2) The scheduling of the sensors in a group is done by designing an easy model as described in the following subsections.

### 3.1. Formation of Groups

**Figure 4:**
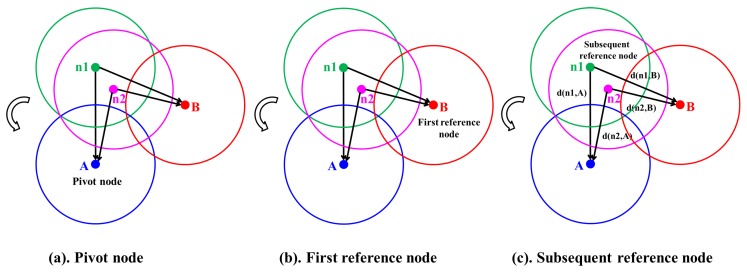
Example of the formation of groups of node A. (**a**) Pivot node selection; (**b**) First reference node selection; (**c**) Subsequent reference node selection.

In this subsection, the formation of groups among sensors is described in which nodes having one-coverage can form a group. First, the pivot node and reference node are selected. The sensor node in a group with the smallest *node_id* is chosen and is designated as a *pivot*node, whereas the neighboring node with least overlapping is designated as the first reference node. As shown in Figure 4a, let us assume that sensor *A* has the smallest *node_id* and is chosen as the pivot node. Among the neighboring nodes (n1, n2 and *B*) of sensor *A*, sensor *B* has the least overlapping region with pivot node *A*. Therefore, sensor *B* is designated as the first reference node as shown in Figure 4b. The subsequent reference nodes are selected by the *pivot* node in increasing order of the overlapping region in an anti-clockwise direction. If the sensing range of any sensor simultaneously overlaps with the first reference node and *pivot* node and at the same time the sum of the distance to them is maximum, then that concerned sensor is chosen as the subsequent reference node. As shown in Figure 4c, the coverage ranges of both sensors *n1* and *n2* simultaneously overlap with the coverage ranges of the *pivot* node (sensor *A*) and the first reference node (sensor *B*). Let us consider that d(n1,A) and d(n1,B) represent the distance from n1 to *A* and *B*, respectively. Similarly, d(n2,A) and d(n2,B) represent the distance from n2 to *A* and *B*, respectively. As shown in Figure 4c, since the sum of the distance from *n1* to *A* and *B* represented as d(n1,A)+d(n1,B) is larger than that of the sensor *n2* represented as d(n2,A)+d(n2,B), the sensor *n1* is designated as the subsequent reference node.

The process of one-coverage initiates from the *pivot* node. The reference nodes are selected and activated by the *pivot* node until its perimeter is fully covered. Once the *pivot* node’s perimeter is fully covered, the role of the *pivot* node is assigned to the first reference node, which in turn selects and activates a group of sensors that covers its perimeter. The process repeats until the monitoring area is fully covered, upon which the chosen sensors are clustered in the same group guaranteeing one-coverage. In PSKGS, the coverage overlapping is reduced by selecting the member sensor of the group based on the distance to the currently-chosen sensors. The aforementioned process of grouping member sensors follows until the set of chosen sensors do not complete the one-coverage. In PSKGS, normally a sensor belongs to one group, and for the sensors that do not fit into any group, they are allowed to go to the sleep state for a short time period.

### 3.2. Scheduling of Nodes

In this sub-section, first, we will present the working schedule structure of a sensor node in a typical WSN followed by the proposed Pre-Scheduling-based K-coverage Group Scheduling (PSKGS) mechanism.

#### 3.2.1. Working Schedule Structure

**Figure 5:**
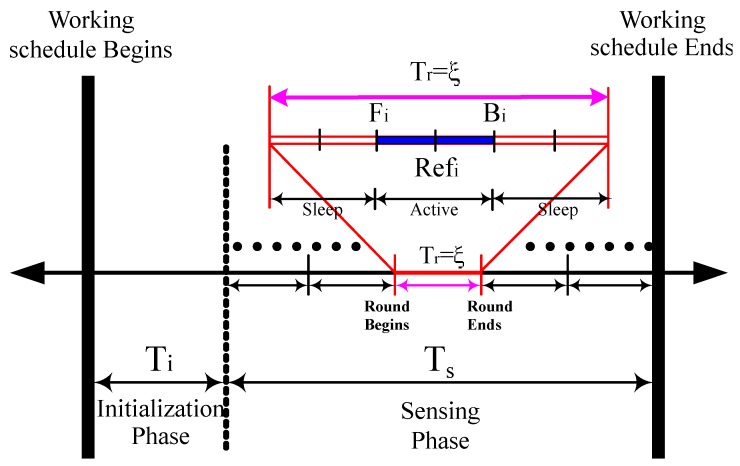
The working schedule of a sensor in WSN.

In a typical WSN, normally each sensor node has a working schedule of the same length, which is subdivided into two disjoint time durations known as the Initialization Phase (IP) represented as $T_{i}$ and the Sensing Phase (SP) represented as $T_{s}$. In Figure 5, an explanation of the working schedule is presented. First, the exchange of information among the neighboring nodes takes place in IP for deriving the working schedules. Later, each sensor node changes its modes between active and sleep in SP based on the working schedule. SP consists of several rounds each with equal time duration represented by $T_{r}$. Before scheduling the nodes, it is essential to derive the mechanism for the calculation of the active period of the sensor nodes.

Let us consider a sensor *i* that randomly selects the reference time $Ref_{i}$ in a round $T_{r}=\xi$. As shown in Figure 5, front ($F_{i}$) and back ($B_{i}$) are defined as the time instances of sensor *i* as the beginning and end of the active period, respectively. The sensor *i* stays active from $F_{i}$ to $B_{i}$ and goes to sleep state for the rest of the time duration. The selection of front ($F_{i}$) and back ($B_{i}$) is very crucial for calculating the proper active period. In Section 3.2.2, the formulas to calculate $F_{i}$ and $B_{i}$ are generalized under different values of coverage-*k*.

#### 3.2.2. PSKGS Mechanism

**Figure 6:**
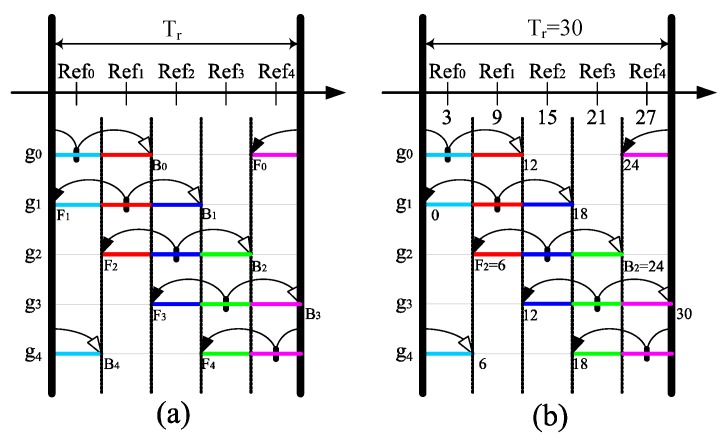
A sleep scheduling in Pre-Scheduling-based *K*-coverage Group Scheduling (PSKGS) for *K* = 3, (**a**) A theoretical representation; (**b**) an example representation.

For constructing the desired *K*-coverage configuration, each member sensor *i* of a one-covered group randomly chooses a reference time (*Ref${}_{i}$*), which is the basis for calculating the working period of a group in each round. Each group *g${}_{i}$* stays active from front (F${}_{i}$) to back (B${}_{i}$) in each round based on its *Ref${}_{i}$* and the required value of *K*. Note that a required *K*-coverage degree cannot exceed the maximum possible coverage of the deployed sensors. Figure 6a illustrates the sleep scheduling of the *r*-th round with the time duration ($T_{r}$) for the coverage degree *K = 3*. In Figure 6a, the deployed sensors are divided into five groups. Here, each group must have at least one member sensor node. For *K* = 3, out of five groups, only three groups need to participate, each with one active sensor to monitor the area at each time segment. To balance the power consumption of the one-covered groups, all reference times are assumed dividing the round $T_{r}$ into equal time durations. PSKGS calculates the working period of each group with a very easy model, which guarantees a required *K*-coverage. For different *K*-coverage requirement (odd and even), a separate formulation is derived for the calculation of front ($F_{i}$) and back ($B_{i}$) as follows.

If *K* is even, F${}_{i}$ and B${}_{i}$ of g${}_{i}$ are calculated as given in Equation (1), where *n* denotes the number of reference times (*Ref*) in a round $T_{r}$.

(1) $$\begin{array}{c} \left\{\begin{array}{cc} F_{i}=Ref_{a}, & if\;a<0,\;where\;\;a=i-\frac{K}{2}\;\;and\;\;a=a+n\\ B_{i}=Ref_{b}, & if\;b\geq n,\;where\;\;b=i+\frac{K}{2}\;\;and\;\;b=b-n \end{array}\right. \end{array}$$

When *K* is odd, g${}_{i}$ works from F${}_{i}$ to B${}_{i}$ as given in Equations (2) and (3), respectively. For c${}_{1}=0$ or d${}_{1}=n-1$, a separate algorithm is designed to calculate F${}_{i}$ and B${}_{i}$ of g${}_{i}$ as given in Algorithm 1.

(2) $$\begin{array}{cc} F_{i}=\frac{Ref_{c_{1}}+Ref_{c_{2}}}{2}, & where\;\left\{\begin{array}{c} c_{1}=i-⎣\frac{K}{2}⎦,\;if\left(c_{1}\;<\;0\right),\;and\;c_{1}=c_{1}+n\\ c_{2}=\left(i-⎣\frac{K}{2}⎦-1\right),\;if\left(c_{2}\;<\;0\right),\;and\;c_{2}=c_{2}+n \end{array}\right. \end{array}$$

(3) $$\begin{array}{cc} B_{i}=\frac{Ref_{d_{1}}+Ref_{d_{2}}}{2}, & \;where\left\{\begin{array}{c} d_{1}=i+⎣\frac{K}{2}⎦,\;if\left(d_{1}\geq n\right),\;and\;d_{1}=d_{1}-n\\ d_{2}=\left(i+⎣\frac{K}{2}⎦+1\right),\;if\left(d_{2}\geq n\right),\;and\;d_{2}=d_{2}-n \end{array}\right. \end{array}$$

 **Algorithm 1: Calculation of $F_{i}$ and $B_{i}$ for the *r*-th round of time duration $T_{r}$.**  **Input**: $c_{1}$, $c_{2}$, $d_{1}$, $d_{2}$, $T_{r}$, $Ref_{c_{1}}$, $Ref_{c_{2}}$, $Ref_{d_{1}}$, $Ref_{d_{2}}$
  **Output**: $F_{i}$, $B_{i}$
 
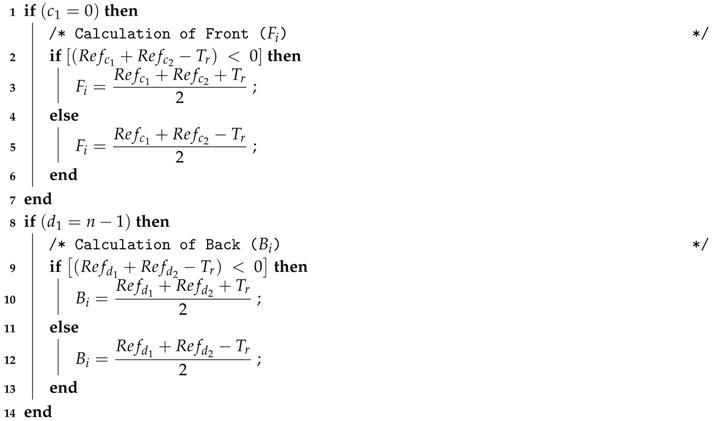


For a group $g_{i}$, if its $F_{i}\leq B_{i}$, then $g_{i}$ stays active for $Len_{i}$, where $Len_{i}=(F_{i}+T_{j}^{B}$, $B_{i}+T_{j}^{B})$. Here, $T_{j}^{B}$ represents the beginning time instance of a round $T_{j}$ and $T_{j}^{B}=T_{j-1}^{B}+T_{j}$, where $T_{j}$ represents the time duration of the round *j* and *j* ≥ 1. Taking group $g_{2}$ in Figure 6a as an example, the $F_{2}$ and $B_{2}$ can be calculated for the odd value of K=3 and round $T_{r}$ as follow. As described in Equation (2), the outcome of $c_{1}$ and $c_{2}$ will be derived to one and zero, respectively. This results in $F_{2}=\frac{Ref_{1}+Ref_{0}}{2}$, which implies that $F_{2}$ begins at the middle of $Ref_{1}$ and $Ref_{2}$ as shown in Figure 6a. Similarly, for $B_{2}$, the values of $d_{1}$ and $d_{2}$ will be calculated using Equation (3), which are three and four, respectively. This gives $B_{2}=\frac{Ref_{3}+Ref_{4}}{2}$, which implies that $B_{2}$ ends at the middle of $Ref_{3}$ and $Ref_{4}$ as shown in Figure 6a. Based on $F_{2}$ and $B_{2}$, the working period of $g_{2}$ is represented as $Len2=(F_{2}+T_{r}^{B},B_{2}+T_{r}^{B})$. To be specific, let us assume that for any *r*-th round beginning at $T_{r}^{B}=0$, the time duration of the round $T_{r}=30$. $T_{r}$ is divided into equal sub-time durations by a set of five reference times ($Ref_{0}$, $Ref_{1}$, $Ref_{2}$, $Ref_{3}$, $Ref_{4}$) separated by 6 s as shown in Figure 6b. As derived earlier for $g_{2}$, the $F_{2}=\frac{Ref_{1}+Ref_{0}}{2}=\frac{9+3}{2}=6$ and $B_{2}=\frac{Ref_{3}+Ref_{4}}{2}=\frac{27+21}{2}=24$. At the beginning of round $T_{r}^{B}=0$, the active period $Len_{2}=(F_{2}+T_{r}^{B}$, $B_{2}+T_{r}^{B})=\left(6+0,24+0\right)=\left(6,24\right)$.

On the other hand, if $F_{i}>B_{i}$, then $Len_{i}=[\left(T_{i}^{B},T_{i}^{B}+B_{i}\right)$ && $\left(T_{i}^{B}+F_{i},T_{i}^{B}+T_{i})\right]$. For example, the working period of $g_{0}$ in Figure 6a has two segments in $T_{r}$. One is from $T_{r}^{B}$ to $\left(T_{r}^{B}+B_{0}\right)$ and the other one is from $\left(T_{r}^{B}+F_{0}\right)$ to $\left(T_{r}^{B}+T_{r}\right)$. To be specific, as shown in Figure 6b, for $T_{r}=30$ and $T_{r}^{B}=0$, the active period $Len_{0}$ of $g_{0}$ is calculated as $Len_{0}=[\left(0,0+12\right)$ && (0+24,0+30)]=[(0,12) && (24,30)].

When all groups establish the duty cycle, each group (g${}_{i}$) periodically wakes up at $F_{i}$ of each round and works for $Len_{i}$. It is to be noted that as time goes by, a few sensors may cease working and go down silently. Normally, each of the active sensors sends a periodical heartbeat message to the neighbors. In cases where the failure of any one of the neighbors is detected by the sensor, it sends a wake up signal to all of the neighbors of the failed sensor to re-schedule the subsequent duty cycles. In the proposed PSKGS algorithm, the network system lifetime is enhanced compared to those of [4,5] as the computational cost of performing the scheduling on grouped sensors reduces effectively.

### 3.3. Computational Complexity of PSKGS

The computational complexity analysis of PSKGS consists of two disjoint parts: (1) the computational cost for the formation of groups; (2) the computational cost for scheduling of nodes. The computational complexity analysis for both parts is described as follows.

#### 3.3.1. Computational Complexity of Forming Groups

Let us assume that a total *N* number of sensors is deployed over the monitoring region. As described in Section 3.1, firstly, the node with the smallest node_id is selected as the pivotnode. The scanning of *N* nodes to select the node with smallest node_id can be accomplished in O(N) time. Next, the pivot node selects the subsequent reference nodes. Let us assume that there are *M* number of neighbors of a pivot node. For the selection of subsequent reference nodes, the information of coverage overlapping is obtained from all *M* neighbors, and it is sorted in the increasing order, which can be accomplished in O(MlogM) time. The subsequent nodes are selected in the increasing order of their coverage overlapping. In summary, the computational complexity of pivot node selection is O(N) and of subsequent nodes selection is O(MlogM). Since M≪N, the combined computational complexity of pivot node and subsequent nodes’ selection is O(N). The aforementioned process of pivot node and subsequent nodes’ selection is repeated for each of the remaining (N−1) nodes. Hence, the total time complexity of the group formation process can be calculated as $O(N^{2})$. However, in PSKGS, the group formation is a one-time process, which is performed just after the deployment of nodes. Hence, only the computational complexity of the scheduling of nodes to evaluate the overall performance should be done as described below.

#### 3.3.2. Computational Complexity of Node Scheduling

In PSKGS, the nodes are scheduled in groups. Let us assume that ζ number of groups is formed out of *N* number of nodes during the group formation process as described in Section 3.1. Any group $g_{l}$ is comprised of *l* number of nodes. For the scheduling of nodes, each node *i* in group $g_{l}$ first randomly selects the reference time instance $Ref_{i}$, which can be accomplished in O(1) time. Later, the value of front ($F_{i}$) and back ($B_{i}$) is calculated for each node based on the $Ref_{i}$ using Equations (1)–(3). Prior to calculation of $F_{i}$ and $B_{i}$, the list of *l* reference times in a group $g_{l}$ is sorted, which can be accomplished in O(llogl) time. In the worst-case scenario, the maximum size of any group can be l=N nodes, when *N* number of nodes is deployed. This results in the worst case time complexity of scheduling *N* nodes, which is O(NlogN).

## 4. Self-Organized-Based *K*-Coverage Scheduling Algorithm

In self-schedule-based *K*-coverage scheduling, the primary goal is to decide whether to keep the sensor in active mode or in sleep mode considering the neighboring nodes for each sensor. Usually, self-organized approaches decide the schedule (active or sleep) of nodes either by tracing all of the InPts or by tracing the perimeter coverage. However, both of the aforementioned approaches are subject to a high computation requirement under the dense deployment scenario. Hence, a novel self-organized-based *K*-coverage scheduling of nodes is highly essential, which precisely decides the schedule of nodes by considering fewer InPts and thereby reduces the computational requirement to prolong the network lifetime and at the same time maintain the detection quality. In this section, the Self-organized-based *K*-coverage Scheduling Algorithm (SKS) is described. The distinct feature of SKS is to decide and conclude the eligibility of each sensor with low computational cost by examining only the candidate regions lying inside the sensing range of each sensor.

### 4.1. Assumptions and Definitions

Before we describe the algorithm, let us discuss the definitions and assumptions. The location-aware stationary sensors are used for the deployment. The sensing range of each sensor is assumed to be identical, and it is represented as *R*. The transmission range of each sensor is considered double to that of the sensing range in order to preserve the connectivity among sensors, and it is represented as 2R. It is assumed that signal decay may negatively impact the detection performance and hence it is presumed that the points located at the boundary of the sensing range may or may not be detected precisely. It is also assumed that any point *p* is considered not covered by sensor *s*, whenever the distance between *p* and *s* represented as d(s,p) is at least that of the sensing range *R*. The aforementioned assumptions are common to WSNs and in line with the assumptions made by several state-of-the-art studies [5,6,7]. The proposed SKS has a unique characteristic of classifying the neighbor set of each sensor into two groups, called as *R* neighbors, represented as $R_{NBs}$, and R−2R neighbors, represented as $R-2R_{NBs}$, which are defined as below.

- $R_{NBs}$: For any sensor *i*, $R_{NBs}$ is defined as $R_{NBs}\left(i\right)=\{j|j\in N,j\neq i,0<d(i,j)<R\}$.
- $R-2R_{NBs}$: For any sensor *i*, $R-2R_{NBs}$ is defined as *R*-$2R_{NBs}\left(i\right)=\{j|j\in N,j\neq i,R\;\leq \;d(i,j)<2R\}$.

**Figure 7:**
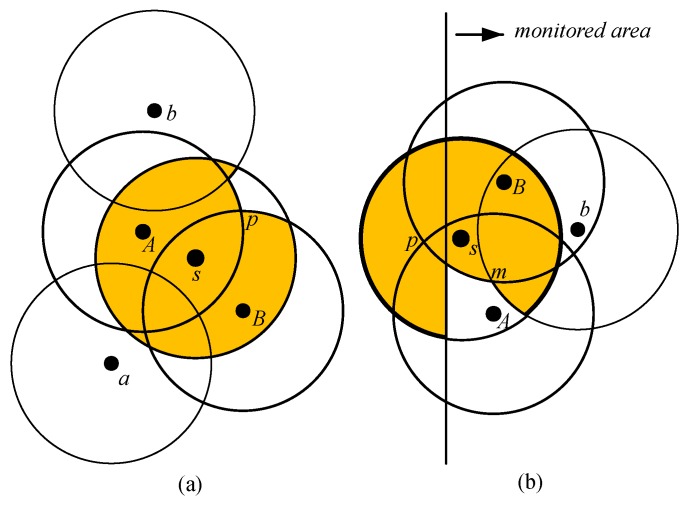
(**a**) The eligibility of *s* is determined by tracing point *p*, which is intersected by $R_{NBs}$, *A* and *B*. (**b**) The intersection point of $R_{NBs}$ is out of the monitored area.

Here, *N* represents the set of sensors located in the monitored region, and *d(i,j)* represents the distance between sensor *i* and sensor *j* For example, as shown in Figure 7a, $R_{NBs}$ of sensor *s* are sensor *A* and sensor *B*, and $R-2R_{NBs}$ of sensor *s* are sensor *a* and sensor *b*. Similarly, as shown in Figure 7b, the $R_{NBs}$ and $R-2R_{NBs}$ set of sensor *s* is {A,B} and {b}, respectively.

### 4.2. SKS Mechanism

**Figure 8:**
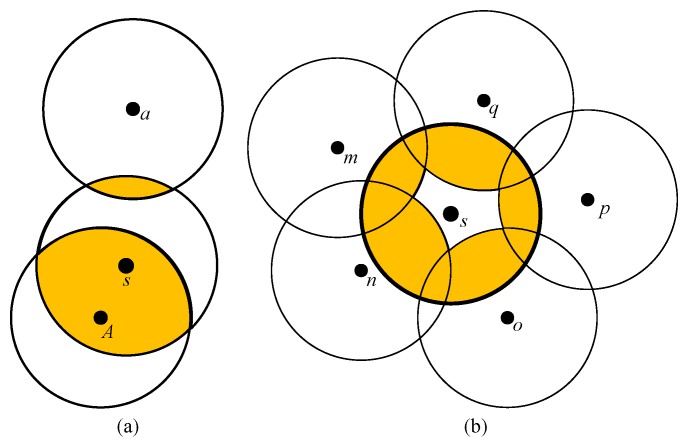
(**a**) For *s*, the area covered by its $R_{NBs}$ (i.e., *A*) is larger than that of its $R-2R_{NBs}$ (i.e., *a*); (**b**) The coverage degree of *s* is 1, if *s* has only $R-2R_{NBs}$.

Based on our scrutiny, there are two explanations for which the neighbor set of any sensor should be classified into $R_{NBs}$ or $R-2R_{NBs}$. The longer the distance d(i,j) of a neighbor j∈N, the lesser the degree of coverage contribution from *j*. For example, as shown in Figure 8a, sensor *A* has a larger overlapping region on the sensing range of sensor *s* compared to that of sensor *a*. Hence, for any sensor *i*, if only $R-2R_{NBs}$ exist, then the coverage degree of sensor *i* can be calculated as one. For example, as shown in Figure 8b, though sensor *s* has many $R-2R_{NBs}$ such as sensors *m*, *n*, *o*, *p* and *q*, the minimum coverage degree within the sensing range of sensor *s* is one. This is because a sensor *s* cannot be fully covered by its $R-2R_{NBs}$ based on our assumption.

Second, the number of $R_{NBs}$ of a sensor is limited to its sensing range. We noticed that for most sensors, the eligibility can easily be decided by considering only the points intersected by their $R_{NBs}$, as depicted in Figure 7a. Taking Figure 7a as an example, sensor *s* has one patch with lower coverage degree within its sensing range, where it is surrounded by the point *p* intersected by sensors *A* and *B*. In this case, sensor *s* just needs to trace the intersection points formed by its $R_{NBs}$. Since the number of $R_{NBs}$ is only 1/3 that of $R-2R_{NBs}$, the computational cost is greatly reduced in many cases, even though the number of deployed sensors is large enough. From the aforementioned analysis, we argue that the characteristics of the $R_{NBs}$ and $R-2R_{NBs}$ of a sensor are worth taking into consideration.

#### 4.2.1. Candidate Regions with Lower Coverage Degree

In several instances, the eligibility of a sensor is decided by SKS by tracing only the InPts of their $R_{NBs}$. It is evident that with the increase in the number of deployed sensors, the complexity of coverage within the sensing range of each sensor also increases. However, SKS robustly decides on the eligibility of sensors by verifying the regions with the lower degree of coverage within their sensing ranges. The sensors under consideration can be categorized into three categories. The first category is known as the edge sensors located near the boundary of the monitoring area. For the edge sensors, the chances are higher for their $R_{NBs}$ InPts to be out of the monitoring area. As shown in Figure 7b, sensor *s* is located at the boundary of the monitoring area, and the point of intersection *p* generated by $R_{NBs}=\left\{A,B\right\}$ of sensor *s* is located outside the monitoring area. Usually, the points intersected by one $R_{NBs}$ and one $R-2R_{NBs}$ form the regions with a lower coverage degree. As shown in Figure 7b, the point of intersection *m* is generated by one $R_{NBs}=\left\{B\right\}$ and one $R-2R_{NBs}=\left\{b\right\}$ of sensor *s* as shown in Figure 7b.

Another category of sensors includes one $R_{NBs}$ and several $R-2R_{NBs}$. Since it is not possible for one $R_{NBs}$ to completely cover the whole sensing range of a sensor, the regions with lower coverage may be surrounded by $R_{NBs}$ and $R-2R_{NBs}$. For that reason, the points intersected by the $R_{NBs}$ and $R-2R_{NBs}$ are traced by SKS. The final and third category is a bit more complex to understand and requires a detailed explanation. Hence, in order to describe the case in detail, several terminologies are used as introduced below.

- Candidate Intersection Points (CIPs): The points intersected by any two $R_{NBs}$ and covered by the fewest $R_{NBs}$.
- Candidate $R_{NBs}$: The $R_{NBs}$, which form the *CIPs*.
- Candidate $R-2R_{NBs}$: The $R-2R_{NBs}$, which cover the *CIPs*.
- Decision points: The points intersected by the *candidate $R_{NBs}$* or *candidate $R-2R_{NBs}$*.

**Figure 9:**
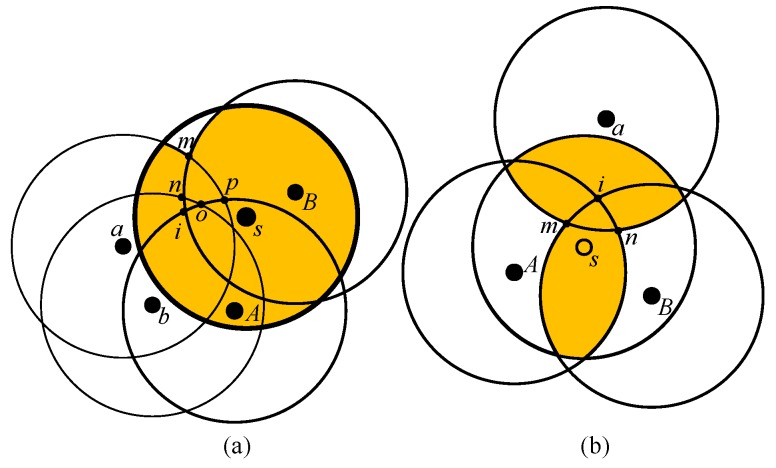
(**a**) The Candidate Intersection Point (*CIP*) denoted as *i* of sensor *s* is covered by *candidate $R-2R_{NBs}$ a* and *b*. (**b**) The points *m* and *n* surrounds the lower coverage regions of *s*.

In the third case scenario, the sensor has few Candidate Intersection Points (*CIP*) covered by several *candidate $R-2R_{NBs}$*. For example, as shown in Figure 9a, sensor *s* has one *CIP* denoted as *i*, which is intersected by $R_{NBs}=\left\{A,B\right\}$ of sensor *s*, and at the same time, it is covered by *candidate $R-2R_{NBs}=\left\{a,b\right\}$* of sensor *s*. By merely tracing a few points such as decision points and CIPs, one can determine the lower coverage of the sensor. Let us consider Figure 9a as an example, where the lower coverage region of sensor *s* surrounded by *m* has the least coverage degree among the *decision points*denoted as *m*, *n*, *o*, *p* and the *CIP* denoted as *i*. This happens as the *candidate $R-2R_{NBs}$* do not cover the lower coverage region completely. A new lower coverage region will be formed by the *candidate $R_{NBs}$* and *candidate $R-2R_{NBs}$*. On the other hand, if the *candidate $R-2R_{NBs}$* cover the region, the lower coverage region will be surrounded by the *candidate $R_{NBs}$* and *candidate $R-2R_{NBs}$* since the coverage contributed by $R-2R_{NBs}$ in the sensing range of a sensor is limited, which are shown as points *m* and *n* in Figure 9b.

Algorithm 2 describes the proposed SKS scheduling in the form of pseudo-code. Initially, all deployed sensors collect the neighbor information. Each sensor divides its neighbor set into $R_{NBs}$ and $R-2R_{NBs}$. If sensor *i* has no $R_{NBs}$(i) (Line 1), its coverage degree is determined as one immediately. This is one of the benefits of classifying the neighbor set of a sensor into two groups. If sensor *i* has no $R-2R_{NBs}$(i) (Lines 2–5), then the coverage degree of *i* is the minimum degree of the *CIPs* within the sensing range of *i* (i.e., A(i)). When *i* has both $R_{NBs}$(i) and $R-2R_{NBs}$(i) (Lines 6–21) and its *CIPs* are not covered by any $R-2R_{NBs}$(i), then the eligibility of *i* is directly determined from the *CIPs* (Lines 17–18). On the other hand, if the *CIPs* are covered by the *candidate $R-2R_{NBs}$(i)*, the algorithm calculates only the coverage degree of the *decision points* intersected by the *candidate $R_{NBs}$(i)* and the *candidate $R-2R_{NBs}$(i)*. In this algorithm, the worst case is that no *CIPs* are found. The algorithm must trace the points intersected by all $R_{NBs}$(i) and $R-2R_{NBs}$(i). Compared to CCP [6], the overall computational cost of SKS is still reduced as the points intersected by any two $R-2R_{NBs}$(i) are not traced. This algorithm terminates when the calculated coverage degree of a sensor is less than *K*.

 **Algorithm 2: Pseudocode of SKS.**
  **Input**: The set of sensors in the monitoring region
  **Output**: Eligible or Ineligible
 
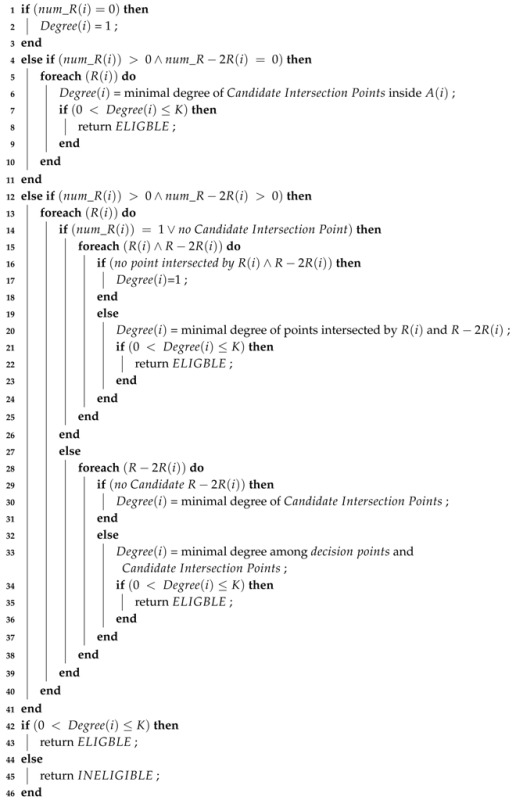


### 4.3. Computational Complexity of SKS

In SKS, the eligibility algorithm presented in Algorithm 2 is executed by each node to decide whether to keep the node active or to change to sleep mode. The fundamental basic step of SKS is to calculate the degree of candidate intersection points inside the sensing range A(i) of each node *i*. Let us assume that for any node *i*, there are α and β number of $R_{NBs}$ and $R-2R_{NBs}$, respectively. There are a total *m* number of intersection points generated by $R_{NBs}$ of sensor *i* that is covered by $R-2R_{NBs}$. If each intersection point can be found with O(t) time complexity, the total time complexity to find *m* candidate intersection points can be calculated as O(m×t). Later, the degree of each candidate intersection point is calculated and stored in a separate list *ℓ*. Finally, a candidate intersection point with a minimal degree is chosen from the list *ℓ*, which can be accomplished with O(m) time complexity by searching each candidate intersection point in a list *ℓ*. The whole aforementioned process is repeated for all of the (N−1) nodes. Hence, the computational complexity of SKS can be calculated as O(m×N×t).

## 5. Performance Evaluation

In this section, the performance of PSKGS and SKS is evaluated using the NS-2 simulator. Besides, our proposed algorithms are compared with several popular pre-scheduled and self-organized algorithms. Through simulation, it is further analyzed to verify which approach maximizes the network system lifetime of the *K*-coverage configuration. In the simulation, all sensors with an identical sensing range of 5 m are deployed over a square region of 50 m × 50 m. For the robust evaluation of the proposed algorithms, nodes are deployed using different random deployment strategies.

### 5.1. Pre-Scheduled Approach

**Figure 10:**
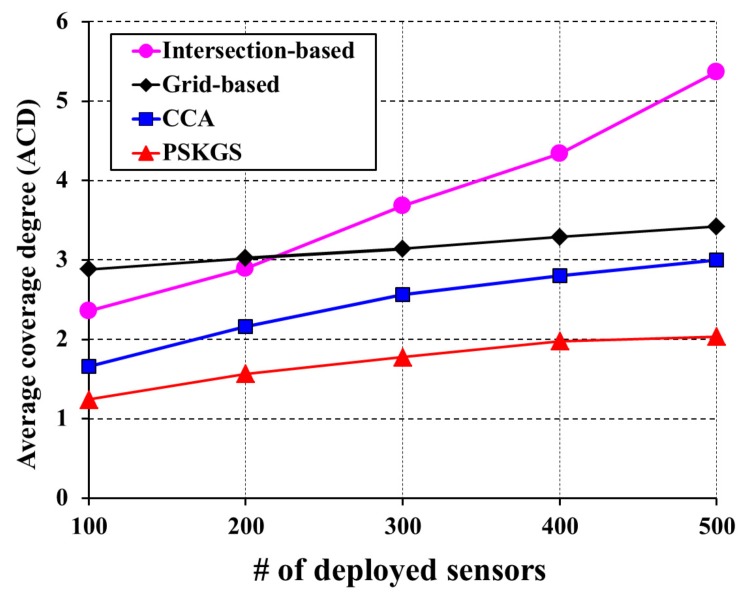
The performance comparison of the intersection-based [4], grid-based [5], Coverage Contribution Area (CCA) [31] and proposed PSKGS with respect to the average coverage degree for *K* = 1.

To verify the performance of our PSKGS algorithm, the state-of-the-art algorithms such as intersection-based [4], grid-based [5] and Coverage Contribution Area (CCA) [31] are implemented for comparison purposes. The intersection-based algorithm [4] is one of the first few pre-scheduled-based scheduling concepts for the degree of *K*-coverage. To maintain the *K*-coverage of the monitoring region, the intersection-based algorithm [4] decides the schedule of the nodes based on the intersection points in a sensing range. Similarly, the grid-based algorithm [5] is also a well-known *K*-coverage scheduling protocol. In the grid-based algorithm [5], scheduling of nodes for *K*-coverage is decided based on the grid points in the sensing range. Since our PSKGS algorithm is designed to propose an alternative method to decide the scheduling of nodes based on the scheduling granularity, its effectiveness can be evaluated by comparing it with the intersection-based [4] and grid-based [5] algorithms. For the synchronization of global time among the sensors, all three aforementioned algorithms perform a lightweight protocol. In the pre-scheduled approach, during the IP, each sensor has to construct its own working schedule, which is later used to carry out the duty cycle in the subsequent long SP. It is known that the system network lifetime of the *K*-coverage configuration is highly dependent on the number of sensors that stay active in each round of the SP. In Figure 10, the performance comparison of the aforementioned algorithms is presented with respect to the average coverage degree in a round. Here, for the coverage, the sensing range of each sensor is divided into 1 m × 1 m virtual GPs in a grid-based algorithm [5]. We define the parameter average coverage degree (ACD) as the number of Active Sensors (ASs) that covers the monitoring area. In order cover the monitoring region with *K* number of sensors, each of the aforementioned algorithms activates a certain number of sensors. From the information of the number of Active Sensors (ASs) and their coverage overlapping, the Average Coverage Degree (ACD) can be derived. The higher the value of ACD, the greater the number of redundant ASs that occur and the performance becomes poorer. Figure 10 presents the outcome of the proposed PSKGS algorithm, intersection-based [4], grid-based [5] and Coverage Contribution Area (CCA) [31] in terms of ACD for K=1. It is observed that for a sparse sensor network, the number of unwanted (redundant) ASs is effectively reduced when applying the intersection-based algorithm [4]. However, with higher node density, the intersection-based algorithm [4] fails to sustain the performance as each sensor has to trace a significantly higher number of intersection points than that required in virtual GPs in a grid-based approach as shown in Table 1. Besides, the Coverage Contribution Area (CCA) [31] achieves lower average coverage degree as compared to intersection-based [4] and grid-based [5], as CCA [31] avoids the selection of sensors whose coverage is already covered by Reuleaux triangles of the neighboring sensors. By selecting the width of Reuleaux triangles, half of the sensing range of a sensor, CCA [31] is able to cover the larger area by choosing *K* sensors for *K*-coverage at the risk of the larger overlapping region from the sensors of adjacent Reuleaux triangles. On the contrary, in our proposed PSKGS algorithm, the sleep schedule is carried out using several groups with each group being one-covered and having the least overlapping among the group members resulting in a reduced Average Coverage Degree (ACD) compared to CCA [31].

**Figure 11:**
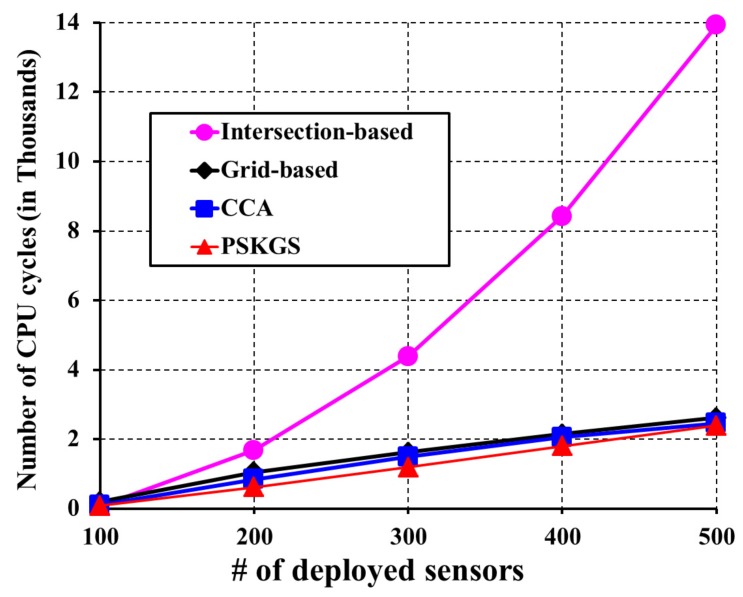
The performance comparison of the intersection-based [4], grid-based [5], CCA [31] and proposed PSKGS with respect to the computational cost for *K* = 3.

It is to be noted that the intersection-based [4] and grid-based [5] algorithms trace a significantly higher number of points, which not only affects the performance to maintain the Average Coverage Degree (ACD), but also simultaneously increases the computational cost of performing a *K*-coverage configuration. The advantage of the PSKGS is the scheduling granularity, which is decided based on the sensing range of a sensor instead of the intersection points or virtual grid points inside it. This results in lowering the average coverage degree of PSKGS as compared to the intersection-based [4] and grid-based [5] algorithms. With the increase in demand for *K*-coverage of the monitoring region, the computational cost requirement also increases to decide the active/sleep scheduling of the nodes. Figure 11 presents the computational cost requirement in terms of the number of CPU cycles for K=3 as the degree of coverage of the monitoring region. From Figure 11, it is also clear that our PSKGS algorithm significantly reduces the computational cost even at high node density compared to the intersection-based algorithm [4] and also shows the marginal reduction as compared to the grid-based [5] and CCA [31]. As mentioned earlier, the intersection-based [4] and grid-based [5] algorithms suffer from a higher computational cost due to the compulsion of tracing a large number of points. On the other hand, in CCA [31], the number of uncovered Reuleaux triangles changes as the coverage process advances, which forces the algorithm to calculate the optimum weight for the sensors and update them from time to time, requiring additional CPU cycles. The CPU cycles are calculated by using Avrora [38], which simulates the aforementioned algorithms executed on MICA2, a widely-used wireless sensor product. The energy model used in Avrora can be briefly described as follow. Depending on the mode, the energy expenditure of the Avrora Mica2 sensor varies. Each Mica2 sensor has broadly three main components such as CPU, radio and EEPROM access. The CPU of a Mica2 sensor can have a set of modes such as active (8.0 mA), idle (3.2 mA), standby (216 μA), power-save (110 μA), power-down (103 μA), extended standby (223 μA), etc. The radio of the Mica2 sensor has primarily two modes: Receiver (Rx) or Transmitter (Tx). Finally, the EEPROM access can have four basic modes such as read (6.2 mA), write (18.4 mA), read time (565 μA) and write time (12.9 ms) [5]. In the pre-scheduled approach, our proposed PSKGS outperforms the state-of-the-art popular algorithms intersection-based [4], grid-based [5], and CCA [31] in terms of the overall coverage degree and power consumption. The detection quality of PSKGS is analyzed later.

### 5.2. Self-Organized Approach

**Figure 12:**
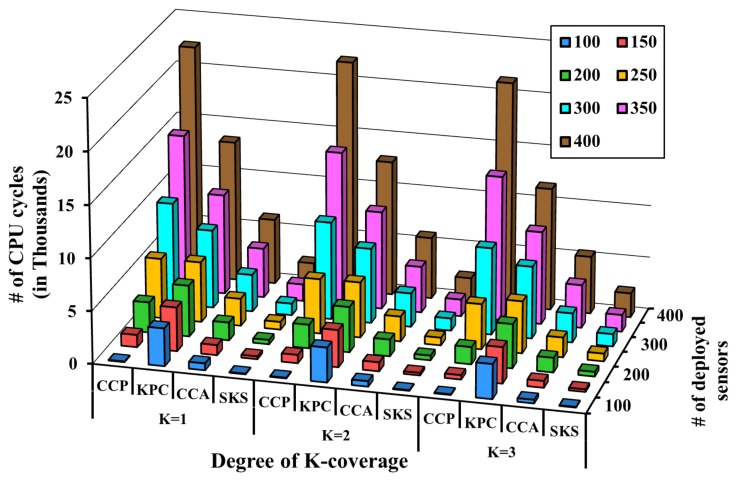
The performance comparison of the Coverage Configuration Protocol (CCP) [6], the *K*-Perimeter-Covered (KPC) algorithm [7], CCA [31] and the proposed Self-Organized *K*-coverage Scheduling (SKS) under different *K*-coverage degrees with respect to the computational cost.

The self-organized approach is different from the pre-scheduled one in that in the self-organized approach, the *K*-coverage eligibility algorithm is executed periodically, and thereby, the maintainability of *K*-coverage primarily depends on the efficiency of the eligibility algorithm. In order to verify the efficiency of the proposed SKS algorithm, two state-of-the-art eligibility algorithms such as the Coverage Configuration Protocol (CCP) [6] and *K*-Perimeter Coverage (KPC) [7] are implemented and compared with it. The Coverage Configuration Protocol (CCP) [6] is the prominent benchmark self-organized *K*-coverage sleep scheduling algorithm. The CCP [6] decides the schedule of the nodes by tracing the intersection points. Similarly, the *K*-Perimeter Coverage (KPC) [7] is also a competent *K*-coverage self-organized scheduling algorithm, where nodes are scheduled by tracing the perimeter of the sensing range. On the other hand, our proposed SKS algorithm is designed to offer an alternative novel *K*-coverage scheduling method by discovering only the candidate region with lower coverage degree within the sensing range of the sensor instead of tracing the intersection points. Hence, to verify the competency of the proposed SKS, it is evaluated against the Coverage Configuration Protocol (CCP) [6] and *K*-Perimeter Coverage (KPC) [7]. The algorithm executing on each sensor terminates as soon as the degree of coverage of a sensor is equal to *K* or less than *K*. In Figure 12, the comparison of the performance outcome of the aforementioned algorithms is shown for K= 1, K= 2 and K= 3 as the degree of coverage. For a thorough evaluation of our protocol and to capture the generalized trend, the algorithms are evaluated under different *K*-coverage requirement with K∈{1,2,3}. All of the algorithms are executed on Avrora under different *K*-coverage configurations. It is to be noted that for the KPC [7], the calculated number of CPU cycles includes the computation, as well as communication cost. This extra communication cost in KPC [7] is incurred due to the inability of KPC [7] to correctly decide on the eligibility of each sensor by tracing only its perimeter coverage. In KPC [7], each sensor must verify the perimeter coverage of all of the neighbors.

It is observed that CCP [6], CCA [31] and our proposed SKS do not incur any extra communication cost and are able to correctly decide the eligibility of each sensor from the information of neighbors as opposed to the KPC [7]. However, CCP [6] has a fundamental drawback of tracing all of the intersection points lying within the sensing range, and CCA [31] requires additional computation power to update the weight of the sensors from time to time. On the other hand, our proposed SKS is less complex and traces only those InPts that are surrounding the regions with lower coverage to correctly decide on the eligibility of each sensor. This effectively suppresses the computational cost of SKS even at higher node density. When 400 sensors are deployed for *K* = 1, the computational cost of SKS is only 11% that of CCP [6]. Besides, since the complexity of SKS is not directly affected by the required coverage degree, i.e., the value of *K*, but by the candidate regions, the complexity of SKS under different values of K∈{1,2,3} in Figure 12 is almost the same. Thus, SKS is scalable in terms of the network size, as well.

### 5.3. Detection Quality and Network Lifetime Evaluation

The proposed PSKGS and SKS adequately improve the performance of the pre-scheduled and the self-organized approach, respectively. However, the improvement of network lifetime should not come at the cost of reduced detection quality of the *K*-coverage configuration. Hence, PSKGS and SKS are further analyzed to verify their ability to sustain the detection quality while maximizing the network lifetime.

#### 5.3.1. Detection Quality

**Figure 13:**
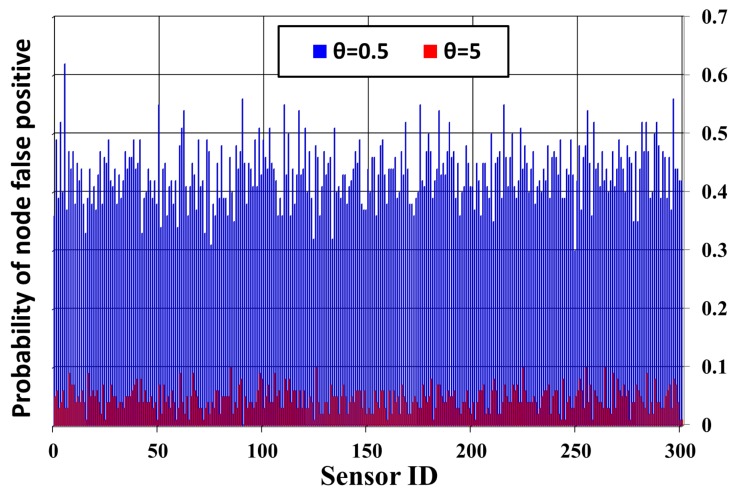
The probability of node false positive of 300 sensors under different detection threshold θ.

In a monitored area, the sensing results obtained from sensors are always accompanied by noise in reality, as shown in Figure 13. Each sensor usually predefines a decision threshold θ on the received signal strength to determine whether an event appears within its sensing range or not. Figure 13 shows that the higher the value of θ, the more noise cases are filtered. A false positive (false alarm) occurs when a sensor receives the signal strength, coming from noise actually, higher than the predefined threshold θ. A false negative (detection miss) arises when an event is present in the sensing range of a sensor, but this sensor does not get any signal strength higher than θ. Several works indicate that the probability of system false positives and the probability of system false negatives are two major performance metrics of WSNs on detection.

**Figure 14:**
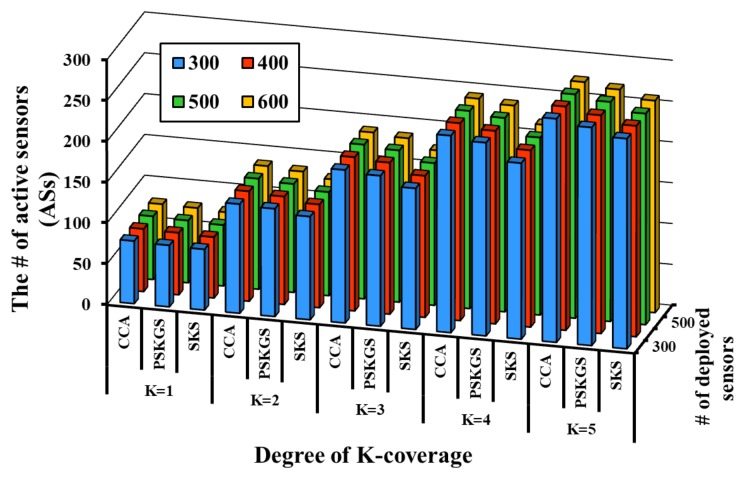
The # of Active Sensors (ASs) in CCA [31], PSKGS and SKS in a round.

**Figure 15:**
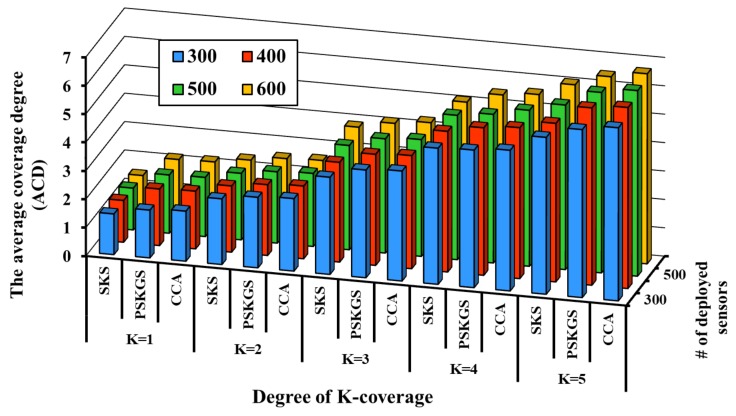
The Average Coverage Degree (ACD) of SKS, PSKGS and CCA [31] in a round.

To improve the detection quality, the authors in [8] define an appropriate value of θ for sensors. However, it is very difficult to define a perfect threshold for each sensor in advance, especially for the sensors that are deployed in a harsh environment. However, the desired detection quality can be guaranteed by performing a data aggregation scheme on the *K*-coverage configuration. The prerequisite here is that the sleep scheduling performed on the *K*-coverage configuration should minimize the number of active sensors while preserving the coverage degree. The detection quality of the proposed PSKGS and SKS is evaluated with respect to CCA [31]. To evaluate the detection quality of PSKGS, SKS and CCA [31], the number of generated ASs is calculated. Figure 14 and Figure 15 show that SKS reduces a greater number of redundant ASs than PSKGS and CCA [31] under the different *K*-coverage degree requirements. Different from SKS, PSKGS establishes the duty cycles of all sensors in the beginning so that some sensors may not work or sleep at the best moment, which backfires and negatively reacts to the detection quality of PSKGS. On the other hand, to cover the Reuleaux triangle with *K* numbers of sensors, the selected sensors in CCA [31] also cover the neighboring Reuleaux triangles. Thus, it enhances the coverage overlapping, leading to the higher chances of severe signal interferences and thereby reducing the detection quality.

**Figure 16:**
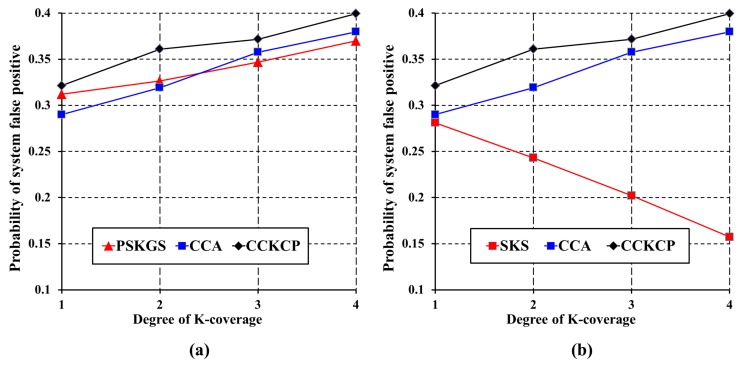
Probability of system false positives of (**a**) PSKGS, CCA [31] and Centralized and Clustered *K*-Coverage Protocol (CCKCP) [32] and (**b**) SKS, CCA [31] and CCKCP [32], with the detection threshold θ=3 and the data aggregation ratio δ=0.3.

**Figure 17:**
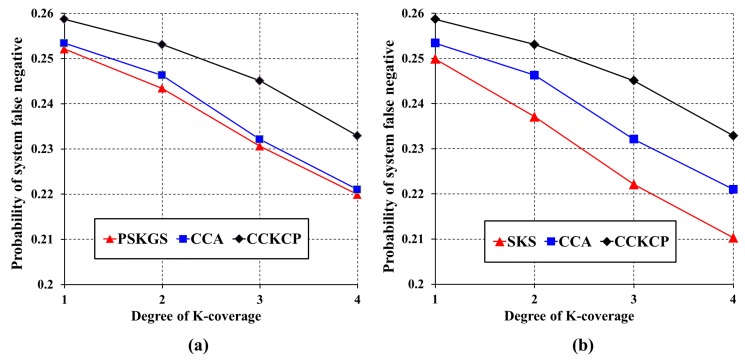
Probability of system false negatives of (**a**) PSKGS, CCA [31] and CCKCP [32] and (**b**) SKS, CCA [31] and CCKCP [32], with the detection threshold θ=3 and the data aggregation ratio δ=0.5.

For the extensive evaluation of the detection quality of the proposed PSKGS and SKS against the probability of the system false positives and system false negatives, an additional state-of-the-art Centralized and Clustered *K*-Coverage Protocol (CCKCP) [32] is included in addition to CCA [31]. Figure 16 shows that our proposed PSKGS has a much higher probability of system false positives than that of SKS under different *K*-coverage configurations. However, at the same time, PSKGS achieves a consistently lower probability of system false positive than CCKCP [32] and produces comparable results to CCA [31] as shown in Figure 16a. This happens as PSKGS generates many redundant ASs as compared to SKS, which is less as compared to CCKCP [32]. Since the CCKCP [32] approach is also based on Reuleaux triangles, it generates a greater number of redundant sensors than CCA [31], as sensors located outside the triangle also contribute to the coverage of the Reuleaux triangle. It is easier for PSKGS to collect sufficient detection results before sending the result to the data center. The reason also causes PSKGS to have a lower probability of system false negatives than that of CCA [31] and CCKCP [32]. On the other hand, if PSKGS wants to maintain the same probability of system false positives as that of SKS by increasing the data aggregation ratio (δ), PSKGS can achieve a higher probability of system false negatives than that of SKS. However, still, it can maintain the lower probability of system false negatives consistently as compared to CCA [31] and CCKCP [32], as shown in Figure 17a. Generally, SKS and PSKGS outperform CCA [31] and CCKCP [32] in terms of detection quality as shown in Figure 17a,b. SKS and PSKGS minimize the number of ASs on the *K*-coverage configuration while guaranteeing the coverage degree.

#### 5.3.2. Network Lifetime

**Figure 18:**
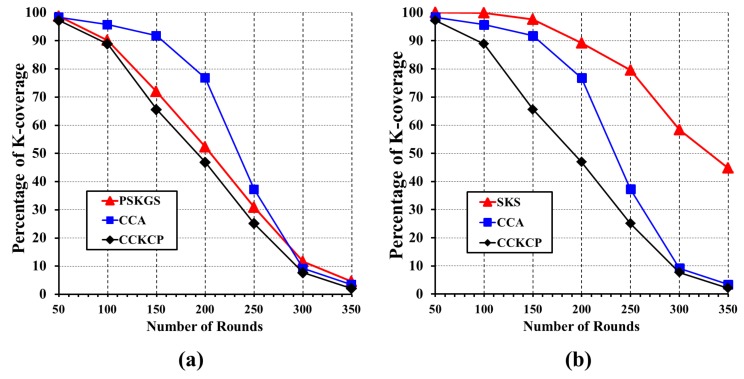
The network lifetime comparison of (**a**) PSKGS, CCA [31] and CCKCP [32] and (**b**) SKS, CCA [31] and CCKCP [32].

The network lifetime performance of SKS and PSKGS is evaluated with respect to CCA [31] and CCKCP [32]. In order to thoroughly evaluate the network lifetime, the number of execution rounds is considered from the time sensors are deployed until 50% of the monitoring area is under *K*-coverage. The energy model of [39] is used to calculate the energy consumption of each sensor. From Figure 18a, it is clear that PSKGS consistently outperforms CCKCP [32] and preserves the *K*-coverage configuration for a longer duration. However, as compared to CCA [31], PSKGS reports the poor performance in the initial rounds and gradually catches up with the performance of CCA [31] as the number of rounds increases. On the other hand, SKS reports consistently superior performance as compared to CCA [31] and CCKCP [32] as shown in Figure 18b. As compared to SKS, PSKGS does incur little cost while maintaining the configuration, as PSKGS does not enforce any eligibility algorithm periodically resulting in blindly following of duty cycles generated in IP by each sensor. However, the pre-scheduled approach like PSKGS does incur a significant extra cost to tackle issues such as fault tolerance. For example, malfunctioning or failure of a single sensor forces all of its neighbors to update their respective duty cycle schedule. Since more and more sensors cease working as time goes by, a significant number of sensors has to re-build the sleep schedule repeatedly. Hence, a rapid drop in the percentage of *K*-coverage is observed in PSKGS. On the other hand, SKS manages the fault tolerance issue by deciding on the eligibility of each sensor based on the remaining energy and thereby it reduces the chances of considering faulty sensors. Moreover, SKS incurs only an insignificant amount of extra cost to perform the eligibility algorithm, in spite of each sensor having to execute the eligibility algorithm periodically. Hence, SKS preserves more energy and thereby enhances the network lifetime compared to PSKGS.

## 6. Conclusions

In the article, at first, various pre-scheduled and self-organized *K*-coverage configuration-based sleep-scheduling algorithms were examined. In the current investigation, our threefold contributions can be described as follows. (1) The proposed PSKGS algorithm demonstrates that the number of unwanted active sensors can be greatly reduced by embedding an effective and suitable node scheduling mechanism in the pre-scheduled approaches. The outcome of the reduction in the number of unwanted active sensors due to appropriate scheduling based on the PSKGS approach resulted in the improvement of the network lifetime. (2) The SKS algorithm is proposed, which accurately determines the eligibility of each sensor by intelligently tracing only the selected important InPts and thereby achieving a noticeable reduction in the computation cost to as low as only 11% compared to the state-of-the-art algorithms under the self-organized approach. (3) The thorough analysis of the results obtained via detailed simulation confirms that in most cases, SKS has superior performance over PSKGS due to the advantage of SKS in the self-organized approach. Such results are useful to select an appropriate sleep-scheduling approach for the *K*-coverage configuration so that the network lifetime of the system can be improved and, at the same time, the proper detection quality is also maintained.

## Figures and Tables

**Table 1:** The average number of Intersection Points (InPts) and GPs traced by each sensor, under the condition of 100~500 deployed sensors.

	100	200	300	400	500
**Intersection-based** [4]	32.68	125.44	269.71	489.66	770.29
**Grid-based** [5]	73.11	73.35	73.41	73.68	73.93
